# Antifatigue Activity and Exercise Performance of Phenolic-Rich Extracts from *Calendula officinalis*, *Ribes nigrum*, and *Vaccinium myrtillus*

**DOI:** 10.3390/nu11081715

**Published:** 2019-07-25

**Authors:** Yu-Tang Tung, Ming-Fang Wu, Mon-Chien Lee, Jyh-Horng Wu, Chi-Chang Huang, Wen-Ching Huang

**Affiliations:** 1 Graduate Institute of Metabolism and Obesity Sciences, Taipei Medical University, Taipei City 11031, Taiwan; 2 Nutrition Research Center, Taipei Medical University Hospital, Taipei City 11031, Taiwan; 3 Graduate Institute of Sports Science, National Taiwan Sport University, Taoyuan 33301, Taiwan; 4 Department of Forestry, National Chung Hsing University, Taichung 402, Taiwan; 5 Department of Exercise and Health Science, National Taipei University of Nursing and Health Sciences, Taipei 11219, Taiwan

**Keywords:** antifatigue activity, antioxidant, polyphenol, salidroside, exercise adaption

## Abstract

*Calendula officinalis*, *Ribes nigrum*, and *Vaccinium myrtillus* (CRV) possess a high phenolic compound content with excellent antioxidant activity. Dietary antioxidants can reduce exercise-induced oxidative stress. Consumption of large amounts of phenolic compounds is positively correlated with reduction in exercise-induced muscle damage. Research for natural products to improve exercise capacity, relieve fatigue, and accelerate fatigue alleviation is ongoing. Here, CRV containing a large total phenolic content (13.4 mg/g of CRV) demonstrated antioxidant activity. Ultra-performance liquid chromatography quantification revealed 1.95 ± 0.02 mg of salidroside in 1 g of CRV. In the current study, CRV were administered to mice for five weeks, and the antifatigue effect of CRV was evaluated using the forelimb grip strength test; weight-loaded swimming test; and measurement of fatigue-related biochemical indicators, such as blood lactate, ammonia, glucose, blood urea nitrogen (BUN), and creatine kinase (CK) activity; and muscle and liver glycogen content. The results indicated that in CRV-treated mice, the forelimb grip strength significantly increased; weight-loaded swimming time prolonged; their lactate, ammonia, BUN, and CK activity decreased, and muscle and liver glucose and glycogen content increased compared with the vehicle group. Thus, CRV have antifatigue activity and can increase exercise tolerance.

## 1. Introduction

Fatigue is one of the most common physiological reactions. The main physiological effect of fatigue is on the energy metabolism during muscle activity [1]. Endurance exercise has a positive effect on antifatigue activity [1]. Currently, scientists are seeking natural products that can not only improve exercise capacity, but also relieve fatigue and accelerate fatigue alleviation without side effects [2]. Fatigue is the loss of normal performance during an athletic activity or exercise and is often associated with tissue damage and energy imbalance. Recovery from fatigue depends on various modes of challenge and treatment. However, few studies have investigated the effects of functional plant extracts on exercise performance and antifatigue activity.

*Calendula officinalis* is a popular medicinal plant rich in bioactive phytochemicals such as terpenoids, flavonoids, coumarins, quinones, volatile oil, and carotenoids [3]. *Ribes nigrum* contains vitamin C and a large amount of phenolic compounds [4,5,6,7]. *Vaccinium myrtillus* has numerous health-promoting properties attributable to proanthocyanidins and anthocyanins [8,9,10,11]. In addition, phenolic compounds with high antioxidant capacity are commonly present in edible plants [12]. In this study, *C. officinalis*, *R. nigrum*, and *V. myrtillus* (CRV), all of which contain high levels of phenolic compounds, were used. Moreover, dietary antioxidants can reduce the effects of exercise-induced oxidative stress and improve physiological conditions [13,14,15]. Recent epidemiological studies have demonstrated that the consumption of large amounts of phenolic compounds is positively correlated with the prevention of exercise-induced muscle damage [16]. Therefore, here, we evaluated the effects of CRV on exercise performance fatigue–associated biochemical indicators in mice.

## 2. Materials and Methods

### 2.1. Materials

The supplement was extracted from *Calendula officinalis*, *Ribes nigrum* and *Vaccinium myrtillus* and provided from HOCKSHENG Trading Co., LTD (Taipei City, Taiwan).

### 2.2. Extraction and Isolation

CRV was cut into small pieces and soaked in 40% ethanol at ambient temperature for 2 h for 3 times. The extract was decanted, filtered under vacuum, concentrated in a rotary evaporator, and then lyophilized. The extract was isolated using a Waters Acquity UPLC system (Waters, Prague, Czech Republic) equipped with a photodiode array detector and a 2.1 × 50.0 mm^2^, 1.7-µm-internal-diameter Waters Acquity BEH C18 column. The mobile phase comprised solvent A, ultrapure water and solvent B, methanol. Elution conditions were 0–5 min of 98–98% A to B; 5–8 min of 98–10% A to B (linear gradient); 8–10 min of 10%–10% A to B; 10–11 min of 10–98% A to B (linear gradient); 11–15 min of 98–98% A to B at a flow rate of 0.8 mL/min. The individual peak area corresponding to salidroside set as the index compound in the UPLC profile was determined at the observed maximal absorbance at 223 nm. A standard calibration curve of salidroside was obtained with a series of standard compound concentrations. Quantification of the index compound in CRV was subsequently performed through UPLC analysis. The peak area of the candidate compound in the chromatogram of CRV was defined, and its content in the sample was obtained on the basis of the quantity calculated from the standard calibration curve.

### 2.3. Determination of Total Phenolics

This assay was determined according to the method described by Tung et al. [17]. The total phenolic content was determined according to the Folin–Ciocalteu method, with gallic acid as the standard. CRV were dissolved in methanol mixed with water (50:50, v/v). The solution (500 µL) was mixed with 500 µL of 50% Folin–Ciocalteu reagent. The mixture was kept for 5 min before 1.0 mL of 20% Na_2_CO_3_ was added. After 10 min of incubation at room temperature, the mixture was centrifuged for 8 min (12,000× *g*), and the absorbance of the supernatant was measured at 730 nm. The total phenolic content was expressed as gallic acid equivalent in milligrams per gram sample. Three replicates were made for each test sample.

### 2.4. Antioxidant Activity

The scavenging activity of CRV DPPH radicals was determined. A dose of 10 μL of CRV in DMSO (1, 5, 10, 50, and 100 μg/mL) was mixed with 200 μL of 0.1 mM DPPH–ethanol solution and 90 μL of 50 mM Tris-HCl buffer (pH 7.4). DMSO and (+)-catechin were used as control and positive control, respectively, for this experiment. After 30 min of incubation at room temperature, the reduction of DPPH radicals was measured by reading the absorbance at 517 nm. The inhibition ratio (%) was calculated as follows: % inhibition = [(absorbance of control − absorbance of test sample)/absorbance of control] × 100. Three replicates were made for each test sample.

### 2.5. Animals and Experimental Designs

**Figure 1:**
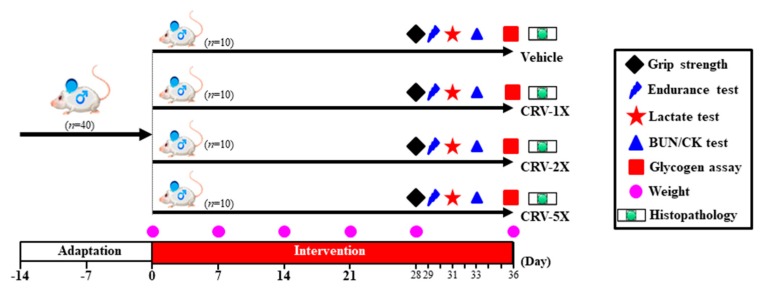
Experimental designs for the effect of *Calendula officinalis*, *Ribes nigrum*, and *Vaccinium myrtillus* (CRV) on antifatigue activity. The animals were randomly assigned to the indicated four groups (vehicle, CRV-1X, CRV-2X, and CRV-5X), and CRV were continual supplemented until the end of the experiment. The physical capacities, related biochemistries, glycogen, and histopathology were assessed in the study.

Male ICR mice (aged 8 weeks) from BioLASCO Taiwan (Yi-Lan, Taiwan) with AAALAC accreditation were used in this study. The standard laboratory diet #5001 (PMI Nutrition International, Brentwood, MO, USA) and distilled water were provided to all animals *ad libitum*, and the environmental conditions were maintained at constant photoperiod, temperature, and humidity (12-h light–12-h dark cycle, 24 °C ± 2 °C and 55–65%, respectively). Routine bedding cleaning was conducted twice per week, and a veterinarian observed and monitored the behavior and health of the animals daily. The Institutional Animal Care and Use Committee (IACUC) of National Taiwan Sport University approved all protocols in the current study with regard to animal welfare considerations, and the study conformed to the guidelines of protocol IACUC-10606 approved by the IACUC Ethics Committee. The starting dosage of CRV supplement recommended was 80 mg/kg, 1×. Furthermore, distinct dependence dosages (vehicle, CRV-1X, CRV-2X, and CRV-5X groups) were designed to validate the possible physiological activities. The detailed experimental procedure is illustrated in Figure 1. All animals were provided with an acclimation period of 2 weeks to adapt to the environment and diet prior to the intervention. The body weight, diet, and social behavior were monitored during supplementation, and daily supplements were administered regularly with fresh CRV sample preparations. Treatment dosages of 0, 80, 160, and 400 mg/kg/day were designated for the vehicle, CRV-1X, CRV-2X, and CRV-5X groups and administered by oral gavage with a volume of 10 mL/kg of the body weight. During 5-weeks CRV treatment, the mice were not subjected to any physical efforts. The grip strength and aerobic endurance capacities were measured to evaluate physical fitness, and the exercise-related biochemistries were immediately assessed during an acute exercise intervention.

### 2.6. Exercise Endurance Performance Test

Exercise performance was based on the survival motives to assess the aerobic endurance capacities. The animals were loaded with a weight equivalent to 5% of their individual body weight and forced to swim in a tank until exhaustion. The persistent time from beginning to exhaustion was recorded as the endurance index. The detailed procedure and protocol were described in our previous study [18].

### 2.7. Forelimb Grip Strength

The grip strength was assessed using a low-force testing system (Model-RX-5, Aikoh Engineering, Nagoya, Japan) for forelimb muscle strength. The details of the method were described in our previous study [19].

### 2.8. Determination of Fatigue-Associated Biochemical Variables

According to our previous report [20], the effect of CRV supplementation on fatigue-related biochemical indicators has been slightly modified to accurately display the physiological status. The animals were fasting for at least 10 h to maintain basal physiological levels before the test. For the lactate metabolite profile, the blood sampling time points were before exercise, immediately after 10 min of acute exercise, and after 20 min of rest. In addition, the NH_3_ and glucose concentrations were analyzed immediately after 10 min of acute exercise. Blood urea nitrogen (BUN) concentration and creatine kinase (CK) activity were subsequently assessed immediately at the 60-min rest time point after 90 min of acute exercise. The blood samples were kept at room temperature for 40 min to enable clotting, after which the samples were centrifuged at 1000× *g* at 4 °C for 15 min. Subsequently, the serum was determined using an AutoAnalyzer (Hitachi 7060, Hitachi, Tokyo, Japan). Besides, the lactate production rate was calculated as the post-exercise rate divided by the before exercise rate (B/A), and the lactate difference between the post-exercise rate and the post-rest rate divided by the post-rest rate was defined as the clearance rate.

### 2.9. Clinical Biochemical Profiles

CRV supplementation was administered continuously until the animals were sacrificed. All mice were euthanatized using 95% CO_2_ asphyxiation 1 h after the last treatment, and blood was immediately sampled by cardiac puncture. Serum was separated through centrifugation, and clinical biochemical variables, including aspartate aminotransferase, alanine transaminase, CK, glucose, creatinine, BUN, UA, total cholesterol, triglyceride, albumin, and total protein levels, were measured using an AutoAnalyzer (Hitachi 7060).

### 2.10. Body Composition and Glycogen Content Analysis

After the mice were sacrificed, the main visceral organs, namely the liver, muscles (gastrocnemius and soleus), kidney, heart, lung, EFP, and BAT, were accurately excised and weighed. The organs were then saved in 10% formalin for further histopathological analysis. Portions of the liver and muscles were stored in liquid nitrogen for glycogen content analysis, as described previously [21].

### 2.11. Histopathology

The visceral organs preserved in 10% formalin were trimmed and embedded in paraffin for tissue sections of 4-μm thickness. Tissue sections were further stained with hematoxylin and eosin and examined under a light microscope equipped with a CCD camera (BX-51, Olympus, Tokyo, Japan) by a veterinary pathologist.

### 2.12. Statistical Analysis

The data were represented as means ± standard errors of the mean (*n* = 10), and the physical activities, biochemistries, body composition, diet, and glycogen content were analyzed through one-way ANOVA for the statistical difference among groups. The Cochran–Armitage test was used to evaluate the dose-effect trend using SPSS (version 19.0). A mixed design two-way ANOVA (supplementation × time) was also applied to the supplementation effects on lactate metabolite profiles and growth curve within repeated time points. Data were considered statistically significant when the probability of a type I error was < 0.05.

## 3. Results

### 3.1. Effect of CRV Supplementation on Grip Strength

**Figure 2:**
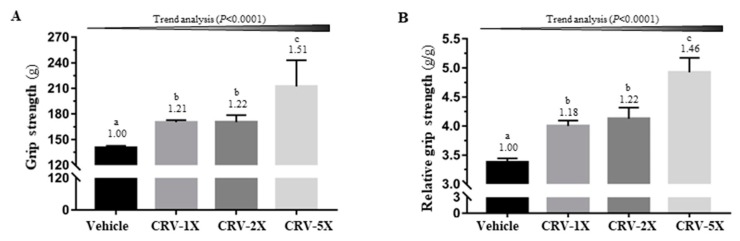
Effect of five-week CRV supplementation on the absolute forelimb grip strength (**A**) and forelimb grip strength relative to the body weight (**B**). Data are expressed as means ± standard errors of the mean for *n* = 10 mice per group. Columns with distinct superscript letters (a, b, and c) differ significantly at *p* < 0.05. Values above the columns are the fold changes compared with the vehicle group.

Figure 2A,B illustrates the difference in absolute and relative grip-strength among groups [F(3, 36) = 20.55 and 14.28, respectively; both *p* < 0.001]. For the supplementation groups, namely CRV-1X, CRV-2X, and CRV-5X, absolute strength was respectively 1.21-, 1.22-, and 1.51-fold and relative strength was 1.18-, 1.22-, and 1.46-fold higher than those of the vehicle group (all *p* < 0.05). In the trend analysis, the absolute grip force and relative grip force of the CRV-treated groups appeared to have significantly increased in a dose-dependent manner (*p* < 0.001).

### 3.2. Effect of CRV Supplementation on Endurance Capacity

**Figure 3:**
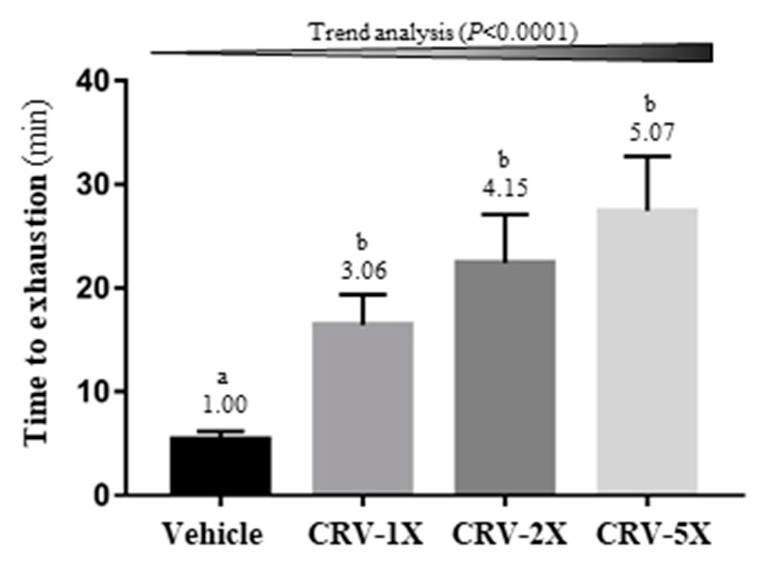
Effect of a five-week CRV supplementation on the exhaustive swimming time. Data are expressed as means ± standard errors of the mean for n = 10 mice per group. Columns with distinct superscript letters (a and b) differ significantly at *p* < 0.05. Values above the columns are the fold changes compared with the vehicle group.

Endurance capacity was measured using the exhaustive swimming test, and the results revealed a significant difference among groups [F(3, 36) = 6.11, *p* = 0.002] (Figure 3). The CRV supplementation groups, namely CRV-1X, CRV-2X, and CRV-5X, had 3.06-, 4.15-, and 5.07-fold higher endurance capacity than the vehicle group; however, endurance capacity did not differ significantly among the CRV supplementation groups. Furthermore, the effects of endurance capacity in the CRV-treated groups significantly increased in a dose-dependent manner (*p* < 0.001).

### 3.3. Effect of CRV Supplementation on Exercise-Related Biochemical Indexes after Exercise Challenge

The lactate metabolite was assessed through repeated measurements (pre-exercise, immediately post-exercise, and after a rest) with various CRV treatments (Table 1). The results indicated significant differences in the supplement main and time effects [F(3, 36) = 27.32 and F(2, 72) = 2932.4, respectively; both *p* < 0.001]. In addition, the difference in the interaction between the supplement main and time effects was significant [F(6, 72) = 174.3, *p* < 0.001]. At the post-exercise and post-rest time points, the lactate concentration among the four groups exhibited significant differences [F(3, 36) = 45.53 and 66.50, respectively; *p* < 0.001]; moreover, the vehicle group had significantly higher lactate concentration than did the CRV-treated groups based on the one-way analysis of variance (ANOVA). In general, CRV supplementation reduced the lactate production rate and increased the clearance rate (*p* < 0.05).

**Figure 4:**
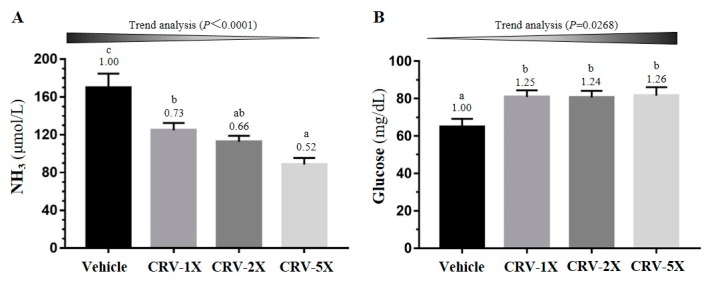
Effect of a five-week CRV supplementation on the serum NH_3_ (**A**) and glucose (**B**) concentrations after acute 10-min swimming exercise challenge. Data are expressed as means ± standard errors of the mean for n = 10 mice per group. Columns with distinct superscript letters (a, b and c) differ significantly at *p* < 0.05. Values above the columns are the fold changes compared with the vehicle group.

The energy homeostasis indexes, ammonia (NH_3_) and glucose concentrations, were measured immediately after the exercise (post-exercise point). As illustrated in Figure 4A, there was a significant difference between the four groups in NH_3_ [F(3, 36) = 12.92, *p* < 0.0001]; moreover, the NH_3_ concentration in the CRV-treated groups significantly decreased by approximately 30–50% compared with that in the vehicle group, all in a dose-dependent manner (*p* < 0.0001). In addition, the difference in glucose between the groups was significant [F(3, 36) = 3.99, *p =* 0.015]; the glucose concentration in the CRV-treated groups significantly increased by approximately 25% (*p* < 0.05; Figure 4B).

**Figure 5:**
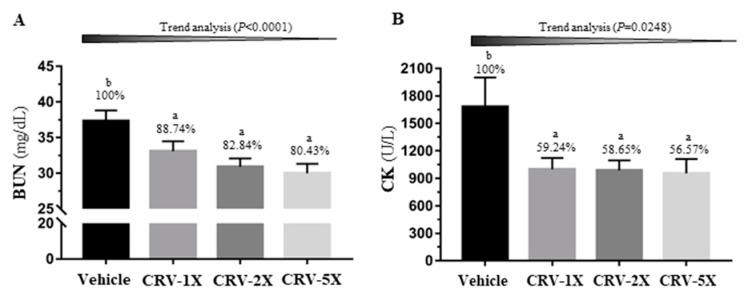
Effect of a five-week CRV supplementation on the serum blood urea nitrogen (BUN) concentration (**A**) and creatine kinase (CK) activity (**B**) after acute exercise challenge. The indicated four groups underwent 90 min of swimming exercise, and blood was sampled after 60 min of rest. Data are expressed as means ± standard errors of the mean for *n* = 10 mice per group. Columns with distinct superscript letters (a and b) differ significantly at *p* < 0.05. Values above the columns are the relative percentage (%) of the vehicle group.

Figure 5A illustrates a significant difference in the other metabolic indicator, blood urea nitrogen (BUN), among groups after acute exercise [F(3, 36) = 5.63, *p =* 0.003]. CRV supplementations significantly decreased the exercise-induced BUN concentration by 12–20% (*p* < 0.05) in a dose-dependent manner (*p* < 0.0001). In addition, the other major injury index creatine kinase (CK) demonstrated significant differences among groups [F(3, 36) = 3.19, *p =* 0.035] (Figure 5B). CRV supplementation alleviated approximately 40% of the increase in CK and exhibited a dose-dependent trend (*p =* 0.0248).

### 3.4. Effect of CRV Supplementation on Glycogen Content

**Figure 6:**
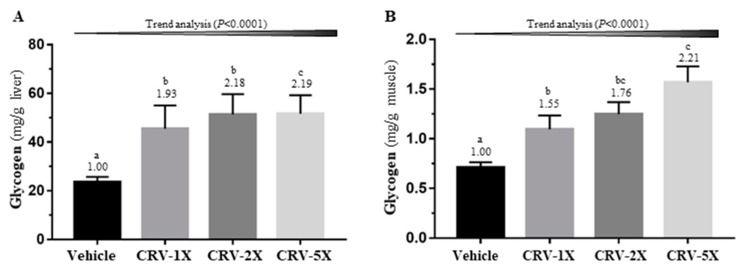
Effect of a five-week CRV supplementation on the liver (**A**) and muscle (**B**) glycogen concentration. Data are expressed as means ± standard errors of the mean for *n* = 10 mice per group. Columns with distinct superscript letters (a, b and c) differ significantly at *p* < 0.05. Values above the columns are the fold changes compared with the vehicle group.

Glycogen stored in the liver and muscle tissue is used for energy regulation and homeostasis. Figure 6A illustrates that CRV supplementation significantly modulated the glycogen content in the liver [F(3, 36) = 3.16, *p =* 0.036]. Compared with the vehicle group, the liver glycogen in the CRV-1X, CRV-2X, and CRV-5X groups increased by 1.93-, 2.18-, and 2.19-fold, respectively, in a dose-dependent manner (*p* < 0.0001). In addition, muscle glycogen concentration exhibited a significant difference among the groups [F(3, 36) = 8.10, *p* < 0.0001]. The muscle glycogen of the CRV-1X, CRV-2X, and CRV-5X groups increased in a dose-dependent manner (*p* < 0.0001) and significantly increased by 1.55-, 1.76-, and 2.21-fold, respectively, compared with that of the vehicle group (Figure 6B).

### 3.5. Subacute Oral Toxicity Evaluation after CRV Supplementation

According to Organization for Economic Co-operation and Development (OECD) Test Guideline 407, subacute oral toxicity evaluation is used to assess the safety of a supplementation. Several indexes including behavior, diet, growth curve, organ weight, biochemistries, and histopathology were evaluated for the subacute toxic effects of CRV supplementation. Behavior was monitored daily during CRV administration, and the behavior was normal among the groups. As presented in Table 2, the treatment main effect did not demonstrate a significant difference in terms of body weight [F(3, 36) = 0.004, *p =* 1.00], but the time main effect exhibited a significant difference in body weight [F(5, 180) = 362.1, *p* < 0.0001]. Therefore, the results indicated that the increase in body weight occurred in a time-dependent manner. However, no significant difference was noted in the interaction effect (supplement × time) [F(15, 180) = 0.279, *p =* 0.997], and the one-way ANOVA at various time points revealed no significant difference between groups (*p* > 0.05). Moreover, no significant differences was noted in the diet and energy intake among CRV-treated groups.

Body composition may also reflect the effect of supplementation on various organs (Table 3). The results indicated that there were no significant differences in the liver, muscle, kidney, heart, lung, epididymal fat pad (EFP), and brown adipose tissue (BAT) among the vehicle and CRV-treated groups (*p* > 0.05), and the relative organ weight adjusted by individual weight revealed similar results. Clinical biochemistry was used to assess the effect of supplementation on physiological conditions. As presented in Table 4, these indicators are related to the liver function, blood lipid, renal function and injury, and metabolic indicators applicable to current biochemical assessments. The uric acid (UA) indexes differed significantly among the groups [F(3, 36) = 11.88, *p* < 0.0001], and they were significantly lower in the CRV-treated groups than in the vehicle group (*p* < 0.05).

**Figure 7:**
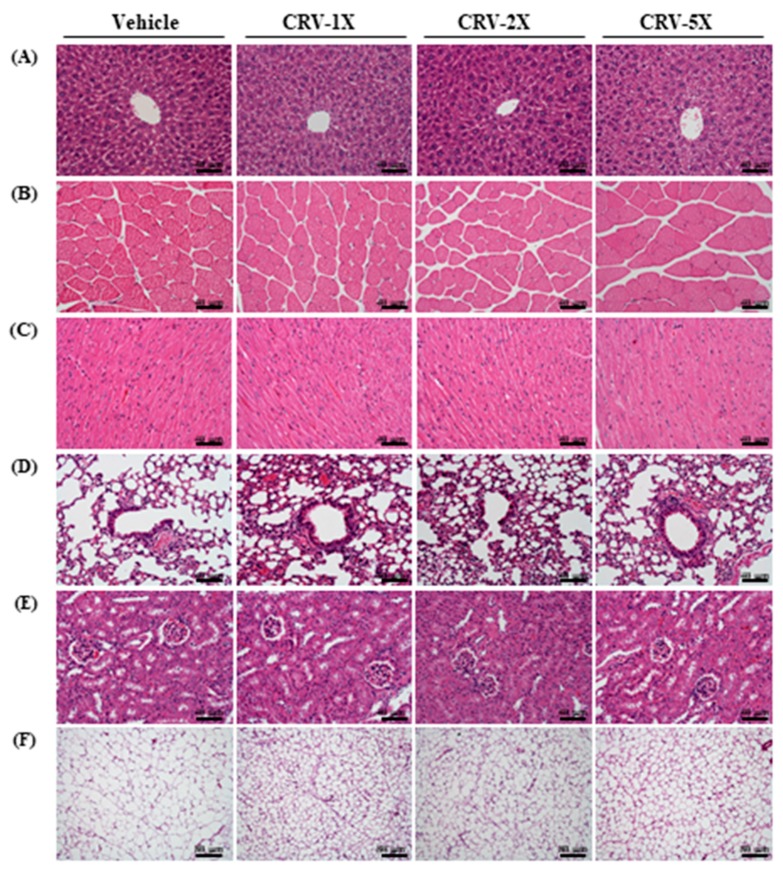
Effect of CRV supplementation on the histomorphological features of the liver (**A**), muscle (**B**), heart (**C**), lung (**D**), kidney (**E**), and adipocyte (**F**) tissue in mice. Specimens were photographed under a light microscope (hematoxylin and eosin staining, magnification: 200×; scale bar, 40 or 80 μm).

Except for UA, no significant differences in biochemical indicators were noted among groups. In addition, the histopathological results did not exhibit pathological abnormalities caused by long-term CRV supplementation, as observed by a clinical veterinarian (Figure 7).

## 4. Discussion

Physical exercise and nutritional behavior are now widely considered major parts of a healthy lifestyle. In addition, moderate exercise and active lifestyle are useful to prevent cardiovascular diseases [22], type 2 diabetes mellitus [23], metabolic syndrome [24], and neurodegenerative diseases [25,26]. Regular and moderate exercise represents a mild source of stress that can induce adaptive responses, and most organisms have the ability to adapt to stress. However, overexercising or overtraining can also lead to muscle damage, oxidative stress, and inflammation. Exercise induces the production of reactive oxygen species, which leads to oxidative stress, such as that by inducing lipid peroxidation [27,28,29,30], activating xanthine oxidase to produce superoxide anion, and increasing the oxidized/reduced glutathione (GSSG/GSH) ratio [31,32]. Moreover, exhaustive exercise can lead to oxidative stress, inflammation, and structural damage to muscle cells, as evidenced by the increased lactate dehydrogenase (LDH) and CK activities [29,33,34]. Therefore, several studies have investigated the possibility of preventing exercise-induced oxidative stress and muscle damage through nutritional interventions [35,36,37,38,39,40,41]. The current study systematically investigated the potential effects of CRV on exercise performance to prevent exercise-induced muscle damage.

*C. officinalis* is a popular medicinal plant and herb used in cosmetics in Europe and the United States. In traditional medicine, its flowers are used in the treatment of various skin conditions such as ulcers, eczema, burns, bruises, rashes, varicose veins, and acne in the form of ointments [42]. *C. officinalis* flower has been reported to possess a wide range of biological activities, including choleretic, anti-inflammatory, analgesic, anticancer, bactericidal, diuretic, tonic [43], anti-HIV, hepatoprotective, spasmolytic, and spasmogenic [3] actions. *C. officinalis* flower extracts are rich in secondary bioactive metabolites such as terpenoids, flavonoids, coumarins, quinones, volatile oil, and carotenoids [3]. Blackcurrant (*R. nigrum*) fruits are a rich source of vitamin C [7], and they contain a large amount of phenolic compounds, including phenolic acids, flavonoids, and most notably anthocyanins (250 mg/100 g of fresh fruit), and have antioxidant activity [4,5,6]. *R. nigrum* has therapeutic benefits in cardiovascular disorder treatment and is commonly reported as a free radical scavenger [44]. Bilberries (*V. myrtillus*) have numerous health-promoting properties attributed mostly to pharmacologically active ingredients (i.e., proanthocyanidins and anthocyanins) [8,9,10,11]. The content of anthocyanins in bilberries represents 60–70% [45]. Bilberries have demonstrated a wide range of biological activities, including antioxidant capacity, astringent and antiseptic properties, ability to reduce the permeability and fragility of capillaries, inhibition of platelet aggregation and urinary tract infection, and strengthening of collagen matrices through cross linkages [46,47,48,49,50].

Recently, plant phenolic compounds have received increased attention because epidemiological studies have revealed that a high phenol intake may positively correlate with the reduction of exercise-induced muscle damage [16]. Therefore, phenolic compounds play a biological and physiological role in the improvement of physical properties. In the current study (data not presented), CRV possessed a large total phenolic content (13.4 mg/g of CRV) and significant DPPH radical scavenging activity (antioxidant activity), with IC_50_ = 38.7 μg/mL. Preethi et al. [51] found that *C. officinalis* flower extract exhibited significant inhibition in DPPH free radical formation with IC_50_ values of 100 μg/mL. Olennikov et al. [52] reported that the basic phenolic groups of total extracts of *C. officinalis* flowers were flavonoids and phenylpropanoids with content values of 10.5 to 46.9 mg/g and 6.1 to 33.5 mg/g, respectively. Bryan-Thomas [53] showed that *R. nigrum* possessed a large total phenolic content (5.7 mg/g) and significant DPPH radical scavenging activity (IC_50_ = 40.8 μg/mL). Saral et al. [54] revealed that *V. myrtillus* had high total polyphenols (11.5-20.7 mg GAE/g dry sample), flavonoids (1. 2-2.7 mg QE/g dry sample) and anthocyanins (3.3-11.5 mg Cyn/g dry sample). Matsunaga et al. [55] found that *V. myrtillus* exhibited radical scavenging ability against DPPH radical, the IC_50_ value being 9.1 μg/mL. CRV is consist of *C. officinalis*, *R. nigrum*, and *V. myrtillus*. Taken together, these results indicate that CRV has better DPPH free radical scavenging ability than *C. officinalis* and *R. nigrum*, but is lower than *V. myrtillus*. In addition, total phenolic compounds of CRV are mainly derived from *C. officinalis* and *V. myrtillus*.

Among three plants of CRV, the major phytocompounds of *C. officinalis* are rutin, quercetin-3-*O*-glucoside, scopoletin-7-*O*-glucoside, isorhamnetin-3-*O*-glucoside and gallic acid [56]. *R. nigrum* is enriched in anthocyanins, delphinidin-3-*O*-glucoside, delphinidin-3-*O*-rutinoside, cyanidin-3-*O*-glucoside, cyanidin-3-*O*-rutinoside, petunidin-3-*O*-rutinoside [57]. In addition, *V. myrtillus* contains delphinidin (the major anthocyanin moiety), sinapic acid (the major phenolic acid in the free form), and p-coumaric acid in the ester, glycoside and ester-bound forms [58]. In this study, 1.95 ± 0.02 mg of salidroside was quantified in 1 g of CRV through ultra-performance liquid chromatography (UPLC). Salidroside has various biological effects, including liver protection [59], antihypoxia activity [60,61], suppression of leukemia cell growth [62], and reduction in the differentiation of 3T3-L1 adipocytes [63]. Salidroside can also stimulate muscle glucose uptake through AMPK activation [64] and alleviate the apoptosis caused by oxidative stress through AKT/mTOR/p70S6K and MAPK pathway regulation [65]. Therefore, these activities may be beneficial to exercise physiological adaption and performance; this result is supported by other experimental validations [66]. Regarding the dose, studies have indicated that relatively high doses (approximately 180 mg/kg) of salidroside can ameliorate exercise fatigue in 1 [67] or 15 [68] days. However, safety remains a major preliminary concern, particularly for single phytocompounds at such high doses. Therefore, the effective dose selected for the antifatigue effect of CRV is 80 mg/kg in the current study, and the safety of the supplementation is defined based on body composition as well as biochemical and pathological observations.

The grip strength and weight-loaded swimming test are commonly used to evaluate muscle fitness and endurance capacities [2,69]. Our results indicated that CRV-treated groups had significantly increased grip strength and prolonged swimming time (Figure 2; Figure 3), suggesting that CRV can improve exercise tolerance in mice. *C. officinalis*-flower and *R. nigrum*-fruit extracts are rich in flavonoids, and studies have proposed that some flavonoids are beneficial to aid exercise and exercise performance [16]. In addition, *R. nigrum* is a rich source of vitamin C. Askari et al. [70] conducted a double-blind clinical trial of 60 male students with an athletic history of at least 3 years and noted that daily treatment with 500 mg of quercetin plus 250 mg of vitamin C for 8 weeks improved some markers, including the lean body mass, basal metabolic rate, and total energy expenditure. Askari et al. [71] also reported that quercetin combined with vitamin C could reduce plasma CK after treadmill exercise. In addition, a study demonstrated that salidroside could significantly upregulate the expression of mTOR, p-mTOR, and myosin heavy chain (MyHC) in muscles and rescue their downregulation induced by inflammatory cytokines [72].

When muscles receive sufficient energy from anaerobic glycolysis during high-intensity exercise, muscles produce large amounts of lactate. Table 1 presents the lactate metabolite of the CRV-treated groups; it significantly decreased after swimming compared with the vehicle group. The results suggest that CRV can decrease the metabolite of blood lactate during exercise. BUN and NH_3_ are key blood biochemical parameters related to fatigue [73]. BUN is the product of energy metabolism during exercise, and it is a sensitive indicator to assess the bearing capability when the human body experiences physical loads. The BUN in vivo and exercise tolerance are positively correlated [74,75,76]. In other words, the body adapts poorly to exercise, the higher is the BUN concentration [77]. Urea is the final product of protein metabolism, mainly formed in the liver. During digestion, proteins are broken down into amino acids. Amino acids contain nitrogen and thus are used to generate energy or other substances needed by the cells; the remaining molecules are excreted as NH_3_. Figure 4A and Figure 5A indicates that the NH_3_ and BUN concentrations of the CRV-treated groups were lower than those of the vehicle group. In addition, high-intensity exercise challenges can cause physical or chemical tissue damage such as sacromeric damage, muscle cell necrosis, and oxidative stress [78]. Therefore, cells release specific proteins, such as CK and myoglobin, into the blood, and these can be used as muscle damage indexes. The CK activity increases with exercise loading. However, the serum CK activity of the CRV-treated groups was significantly lower than that of the vehicle group (Figure 6B). CRV extracts contain not only phenolic compounds, but also salidroside that exerts protective effects against the oxidative stress induced by free radicals [79]. Therefore, CRV also ameliorates the stress and fatigue. Energy during exercise is originally derived from the breakdown of glycogen, followed by the circulation of glucose released from the liver [80]. Therefore, liver and muscle glycogen are sensitive parameters related to fatigue. Figure 7A,B illustrates that the liver and muscle glycogen concentration of the CRV-treated groups were higher than those of the vehicle group after swimming. The glycogen is an important energy source to maintain the energy metabolism during physical activity and the exercise training or nutritional supplement could also modulate the glycogenolysis during exercise [81,82]. Besides, the other study also showed the glycogen content could be elevated by nutritional supplement for better performance [83,84]. Thus, the CRV could also positively regulate the glycogen metabolism for the higher exercise performance in current study. The effects of the animal experiment may not be extrapolated to clinical trials because of diversity on physiological and genomic backgrounds. However, the animal studies could not only explore the physiological effects from basic research point view, but also reveal the potential side effects regarding to toxicological and pathological evaluation and observation.

## 5. Conclusions

CRV has significant antifatigue effects, and these effects occur in a dose-dependent manner. Moreover, the possible antifatigue mechanisms reduced the concentrations of NH_3_ and BUN, and CK activity; increased glucose concentration; increased liver and muscle glycogen concentrations by improving the energy storage; elevated lactate metabolite concentration; and protected the muscle tissue. Therefore, CRV can improve the aerobic and anaerobic exercise capacity and physiological adaption in practical applications. CRV, which are rich in phenolic compounds and salidroside, can be potential supplementation alternatives for fatigue improvement; In addition, we also elucidated the doses of CRV with long-term supplementation were safe based on the observation of pathology, body composition, growth curve, and biochemistry indexes. However, further research for evaluating the antifatigue activity at the molecular levels and clinical trial is warranted.

## Figures and Tables

**Table 1:** Effects of *Calendula officinalis*, *Ribes nigrum*, and *Vaccinium myrtillus* (CRV) on lactate metabolite profiles during acute exercise challenge.

Time Point	Vehicle	CRV-1X	CRV-2X	CRV-5X
	Lactate (mmol/L)			
Before swimming (A)	2.9 ± 0.2 ^a^	2.9 ± 0.2 ^a^	2.9 ± 0.2 ^a^	2.9 ± 0.1 ^a^
After swimming (B)	8.5 ± 0.3 ^b^	5.7 ± 0.2 ^a^	5.5 ± 0.2 ^a^	5.4 ± 0.2 ^a^
After a 20-min rest (C)	6.4 ± 0.2 ^b^	4.0 ± 0.2 ^a^	3.7 ± 0.2 ^a^	3.5 ± 0.2 ^a^
	Rate of lactate production and clearance			
Production rate = B/A	2.96 ± 0.1 ^b^	2.03 ± 0.1 ^a^	1.92 ± 0.07 ^a^	1.85 ± 0.04 ^a^
Clearance rate = (B − C)/B	0.25 ± 0.01 ^b^	0.31 ± 0.02 ^a^	0.32 ± 0.01 ^a^	0.34 ± 0.02 ^a^

The lactate metabolites were assessed for the four groups, namely vehicle, CRV-1X, CRV-2X, and CRV-5X, at three repeated time points within each group. Data are expressed as means ± standard errors of the mean for n = 10 mice per group. Values in the same row with distinct superscript letters (a and b) differ significantly at *p* < 0.05.

**Table 2:** Growth curve and dietary profiles during the experiment.

Time Point	Vehicle	CRV-1X	CRV-2X	CRV-5X
Initial BW (g)	38.2 ± 0.4	38.1 ± 0.8	28.1 ± 0.7	38.2 ± 0.5
1st wk BW (g)	40.0 ± 0.7	40.0 ± 1.0	40.0 ± 0.8	40.0 ± 0.3
2nd wk BW (g)	40.5 ± 0.6	40.8 ± 0.8	40.5 ± 0.8	40.8 ± 0.4
3rd wk BW (g)	41.0 ± 0.7	41.0 ± 1.0	41.0 ± 0.8	41.0 ± 0.3
4th wk BW (g)	41.7 ± 0.6	41.7 ± 1.1	41.7 ± 0.8	41.9 ± 0.2
Final BW (g)	42.7 ± 0.7	42.7 ± 1.1	42.7 ± 1.0	42.7 ± 0.3
**Water intake** (mL/mouse/day)	9.7 ± 0.2	9.6 ± 0.2	9.7 ± 0.2	9.6 ± 0.1
**Chow 5001** (g/mouse/day)	7.1 ± 0.1	7.0 ± 0.1	7.0 ± 0.1	7.1 ± 0.1
**Energy intake** (Kcal/mouse/day)	23.8 ± 0.3	23.6 ± 0.3	23.5 ± 0.3	23.8 ± 0.5

The weight and diet were measured regularly for the four groups namely the vehicle, CRV-1X, CRV-2X, and CRV-5X groups. Data are expressed as means ± standard errors of the mean for *n* = 10 mice per group.

**Table 3:** Effects of CRV on body composition.

Characteristic	Vehicle	CRV-1X	CRV-2X	CRV-5X
Liver (g)	2.27 ± 0.07	2.29 ± 0.10	2.26 ± 0.08	2.25 ± 0.05
Muscle (g)	0.41 ± 0.01	0.41 ± 0.01	0.41 ± 0.01	0.41 ± 0.01
Kidney (g)	0.62 ± 0.02	0.62 ± 0.02	0.62 ± 0.02	0.63 ± 0.02
Heart (g)	0.25 ± 0.01	0.24 ± 0.01	0.24 ± 0.01	0.23 ± 0.01
Lung (g)	0.23 ± 0.01	0.23 ± 0.01	0.23 ± 0.01	0.23 ± 0.01
EFP (g)	0.29 ± 0.04	0.29 ± 0.03	0.29 ± 0.04	0.29 ± 0.02
BAT (g)	0.12 ± 0.003	0.12 ± 0.002	0.12 ± 0.01	0.12 ± 0.01
Relative liver weight (%)	5.30 ± 0.08	5.34 ± 0.12	5.27 ± 0.09	5.28 ± 0.07
Relative muscle weight (%)	0.95 ± 0.01	0.97 ± 0.01	0.97 ± 0.01	0.95 ± 0.01
Relative kidney weight (%)	1.44 ± 0.02	1.45 ± 0.03	1.46 ± 0.02	1.47 ± 0.03
Relative heart weight (%)	0.58 ± 0.01	0.57 ± 0.01	0.56 ± 0.02	0.52 ± 0.01
Relative lung weight (%)	0.54 ± 0.003	0.54 ± 0.01	0.54 ± 0.003	0.53 ± 0.01
Relative EFP weight (%)	0.67 ± 0.08	0.67 ± 0.06	0.67 ± 0.08	0.67 ± 0.05
Relative BAT weight (%)	0.28 ± 0.003	0.29 ± 0.003	0.28 ± 0.01	0.28 ± 0.01

Data are expressed as means ± standard errors of the mean for *n* = 10 mice per group. EFP: epididymal fat pad; BAT: brown adipose tissue.

**Table 4:** Effects of CRV on clinical biochemical analysis at the end of the experiment.

Parameter	Vehicle	CRV-1X	CRV-2X	CRV-5X
AST (U/L)	73 ± 4	68 ± 3	69 ± 5	69 ± 2
ALT (U/L)	49 ± 3	41 ± 2	41 ± 2	42 ± 3
CK (U/L)	199 ± 25	173 ± 23	176 ± 26	176 ± 26
GLU (mg/dL)	149 ± 4	144 ± 3	145 ± 3	144 ± 4
CREA (mg/dL)	0.25 ± 0.01	0.24 ± 0.01	0.23 ± 0.01	0.24 ± 0.01
BUN (mg/dL)	20.5 ± 0.6	20.0 ± 0.5	20.4 ± 0.7	20.1 ± 0.7
UA (mg/dL)	1.2 ± 0.1 ^b^	0.8 ± 0.04 ^a^	0.8 ± 0.1 ^a^	0.7 ± 0.03 ^a^
TC (mg/dL)	145 ± 4	141 ± 5	142 ± 6	135 ± 4
TG (mg/dL)	174 ± 6	157 ± 5	157 ± 8	158 ± 6
ALB (g/dL)	2.9 ± 0.03	2.9 ± 0.03	2.9 ± 0.04	3.0 ± 0.05
TP (g/dL)	4.9 ± 0.1	4.9 ± 0.05	4.9 ± 0.04	4.9 ± 0.04

Data are expressed as means ± standard errors of the mean for *n* = 10 mice per group. Values in the same row with distinct superscript letters (a and b) differ significantly at *p* < 0.05. AST: aspartate aminotransferase; ALT: alanine transaminase; CK: creatine kinase; GLU: glucose; CREA: creatinine; BUN: blood urea nitrogen; UA: uric acid; TC: total cholesterol; TG: triacylglycerol; ALB: albumin; TP: total protein.
